# Insights Into the Role of Platelet Factor 4 in Neurodegenerative Diseases

**DOI:** 10.1002/cns.71118

**Published:** 2026-10-06

**Authors:** Qi Tian, Yanji Tong, Fang Yu, Xiaoming Hu

**Affiliations:** ^1^ Department of Neurology, School of Medicine University of Pittsburgh Pittsburgh Pennsylvania USA; ^2^ Geriatric Research, Education and Clinical Center Veterans Affairs Pittsburgh Health Care System Pittsburgh Pennsylvania USA; ^3^ Department of Neurology Westchester Medical Center New York Medical College Valhalla New York USA

**Keywords:** CXCR3 signaling, immune modulation, neurodegenerative diseases, neuroprotection, platelet factor 4

## Abstract

**Background:**

As population aging accelerates worldwide, the prevalence and burden of neurodegenerative diseases (NDs) continue to rise, underscoring the urgent need to identify key molecular mechanisms driving disease progression and novel therapeutic targets. Chronic neuroinflammation is a hallmark of multiple NDs, including Alzheimer's disease and Parkinson's disease. Platelet factor 4 (PF4) is a chemokine primarily released by activated platelets and has traditionally been investigated for its roles in hemostasis, thrombosis, and peripheral inflammation.

**Results:**

Growing evidence has implicated PF4 in the pathogenesis of NDs. Altered PF4 expression has been observed across multiple NDs. As a central player at the intersection of the nervous, immune, and vascular systems, PF4 modulates inflammation, vascular homeostasis, and neuronal survival, all of which are critical in the pathogenesis of NDs. Dissecting the cell‐type specific and temporally dynamic roles of PF4 across diverse disease contexts is essential for elucidating its contribution to neurodegenerative processes and disease progression.

**Conclusion:**

This review highlights PF4 as a potential mechanistic mediator in NDs and discusses its promise as both a biomarker and a therapeutic target. Further elucidation of PF4 signaling pathways may provide new insights into disease mechanisms and uncover novel opportunities for targeted interventions in NDs.

## Graphical abstract

This review summarizes potential functions of platelet factor 4 (PF4) in neurodegenerative diseases (NDs) and discusses its promise as both a biomarker and a therapeutic target. Further elucidation of PF4 signaling pathways may provide new insights into disease mechanisms and uncover novel opportunities for targeted interventions in NDs.

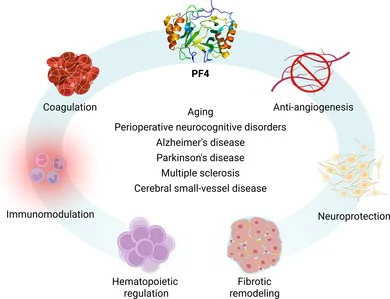

## 1 Introduction

Neurodegenerative diseases (NDs), including Alzheimer's disease (AD) and Parkinson's disease (PD), are chronic disorders characterized by the progressive loss of neurons in the central nervous system (CNS), which leads to motor and/or cognitive deficits [1]. As population aging accelerates worldwide, the incidence and socioeconomic burden of NDs are steadily rising [1]. However, effective interventions capable of delaying, halting, or reversing disease progression remain largely unavailable. Although NDs differ in their clinical manifestations and etiologies, they share several common pathological mechanisms such as chronic neuroinflammation, synaptic dysfunction, mitochondrial damage, and oxidative stress [2]. Among these, neuroinflammation has emerged as a central driver of disease progression [3, 4, 5]. Persistent activation of microglia and astrocytes is commonly observed in neurodegenerative brains, leading to the release of large amounts of pro‐inflammatory cytokines, chemokines, and free radicals. Sustained neuroinflammation can disrupt the blood–brain barrier (BBB), allowing the infiltration of peripheral immune cells into brain parenchyma. The influx of peripheral immune cells further amplifies local inflammatory responses, leading to progressive neuronal damage and neurodegeneration [4]. Understanding the cellular and molecular mechanisms may provide critical insights for the development of effective therapies against NDs.

PF4, also known as chemokine (C‐X‐C motif) ligand 4 (CXCL4), is a CXC chemokine released primarily by activated platelets during coagulation and inflammation [6]. It is a well‐established regulator of vascular biology, contributing to leukocyte recruitment, endothelial activation, angiogenesis, and hemostasis [6]. PF4 is also involved in autoimmune and thromboinflammatory conditions, including heparin‐induced thrombocytopenia (HIT), highlighting its broader immunomodulatory role in systemic inflammation [7]. Emerging evidence suggests that PF4 can also modulate neuroinflammatory responses and influence the activity of brain myeloid cells, including microglia and monocyte‐derived macrophages, thereby shaping the neuroimmune microenvironment [8]. Systemic PF4 administration attenuates hippocampal neuroinflammation, promotes synaptic plasticity–associated molecular programs, and improves cognitive function in aged mice. These findings raise the possibility that PF4 may function as an important, yet underexplored, regulator that links vascular response, immune regulation, and neurodegenerative pathways. However, the precise cellular targets and mechanisms of PF4 signaling in the brain remain to be fully elucidated.

This review aims to systematically summarize current knowledge regarding PF4 expression and function in major NDs—including AD and PD, as well as other neurological disorders associated with secondary neurodegeneration. We discuss the potential mechanisms through which PF4 regulates neuroinflammation, vascular integrity, and neuronal fate, and evaluate its promise as a biomarker and a therapeutic target. We further highlight critical knowledge gaps and future research directions concerning PF4 function in the brain. Uncovering the multifaceted roles of PF4 may provide new insights into neuro‐immune‐vascular interactions and establish a conceptual framework for the development of innovative therapeutic strategies.

## 2 Structure, Expression, and Function of PF4

### 2.1 Structure

PF4 is a highly basic chemokine composed of 70 amino acids with a molecular weight of approximately 7.8 kDa (Figure 1). The PF4 monomer consists of a flexible N‐terminal region carrying the CXC motif, a hydrophobic core containing three antiparallel β‐strands, and a C‐terminal α‐helix. Its high lysine and arginine content creates a highly cationic surface [9]. PF4 can exist as monomers, dimers, and tetramers. Under physiological concentrations, PF4 predominantly forms highly stable homotetramers. Tetramerization of PF4 generates a characteristic positively charged surface (“ring of positive charge”) that facilitates high‐affinity interactions with negatively charged molecules such as heparin and other glycosaminoglycans through electrostatic interactions [6, 10]. The binding of polyanions can also induce conformational rearrangements in PF4 tetramers, altering surface epitope exposure and intermolecular interactions [11]. In addition to homotetramerization, PF4 assembles into heterocomplexes with other chemokines, thereby expanding its biological functions. For instance, PF4‐CXCL8 heterocomplexes inhibit angiogenesis [12]. PF4–CXCL12 heterocomplex suppresses CXCL12‐mediated cell migration by interfering with CXCR4 signaling [13]. PF4–CCL5 heterodimers formed on the vascular endothelium promote monocyte adhesion and recruitment, thereby amplifying vascular inflammation [14]. In addition, PF4, when forming heterodimers with IL‐8, may modulate CXCR2 or glycosaminoglycan–protein interactions, although the biological significance of these mechanisms remains unclear [15]. Like many chemokines, PF4 exhibits receptor promiscuity, whereby a single chemokine can interact with multiple receptors, while individual receptors may recognize several chemokine ligands [16] PF4 has been reported to signal through several chemokine receptors, including CXCR3 [17] and CCR1 [18].

**FIGURE 1:**
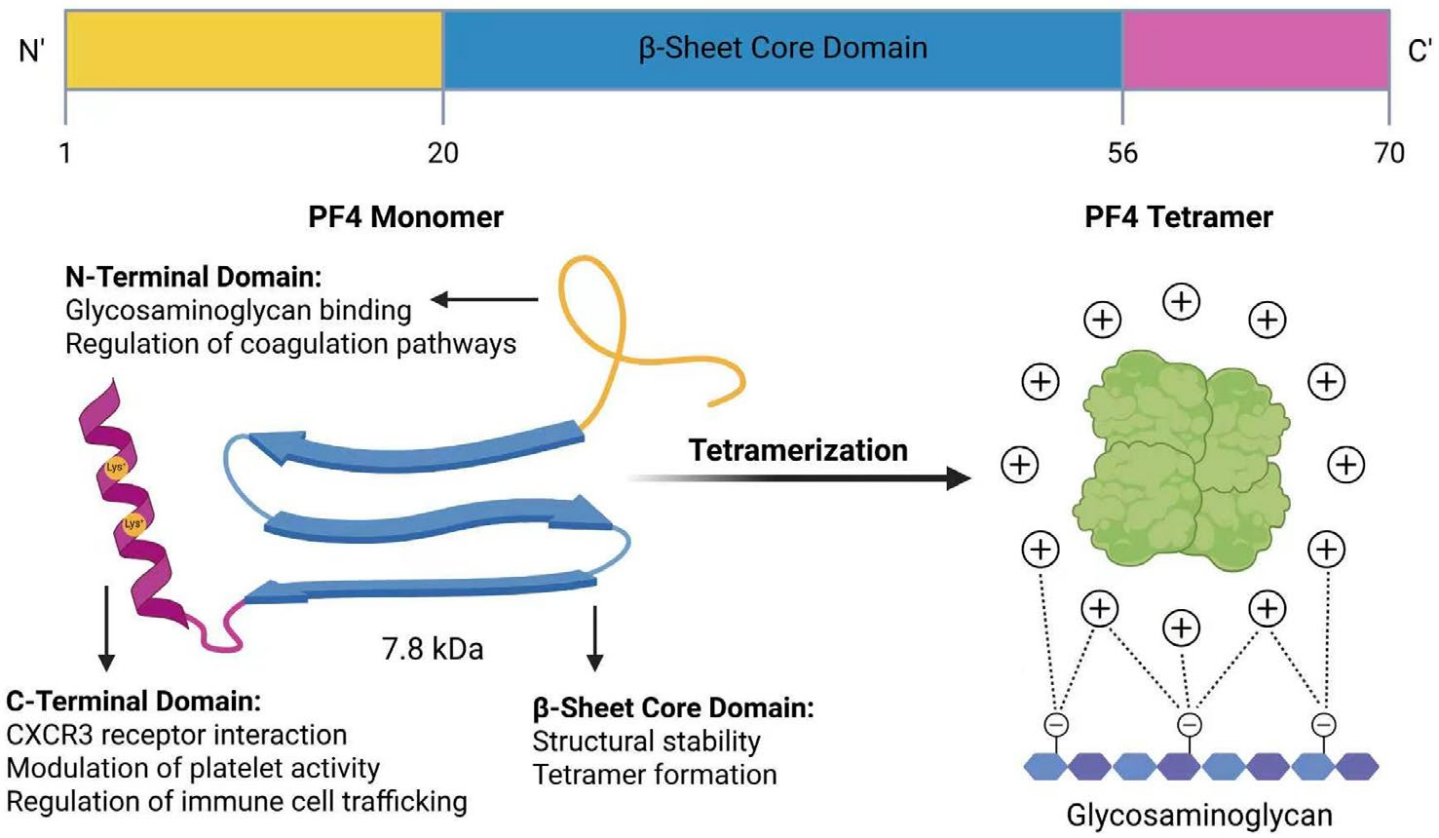
Structural organization, oligomerization, and glycosaminoglycan binding of PF4. PF4 is a highly basic chemokine composed of 70 amino acids and has a molecular weight of approximately 7.8 kDa. The PF4 monomer consists of three structural regions: (1) a relatively flexible N‐terminal region containing a CXC motif; (2) a central structural core composed of three antiparallel β‐strands; and (3) a C‐terminal α‐helix. PF4 exists in a concentration‐dependent equilibrium among monomers, dimers, and tetramers. Upon oligomerization, multiple lysine and arginine residues from adjacent PF4 subunits, including lysine residues within the C‐terminal region, aggregate together to form an extended cationic surface. This cationic surface mediates high‐affinity binding to negatively charged molecules, such as glycosaminoglycans, and plays a critical role in the biological functions of PF4. This figure was generated using BioRender.com.

### 2.2 Expression

PF4 is predominantly synthesized by megakaryocytes and stored in platelet α‐granules [19]. Upon platelet activation by thrombin, collagen, or other agonists, PF4 is rapidly released into the local milieu and circulation, making it one of the hallmarks of platelet degranulation [20]. Each 10^9^ platelets contain approximately 20 μg of PF4. The resting plasma concentrations of PF4 typically range from 2 to 10 ng/mL [9]. More recent studies have demonstrated that PF4 is also expressed in several immune cell populations, including activated monocyte/macrophage [21], activated T cell [22], mast cells [23], and dendritic cells [24]. Within the CNS, PF4 is highly expressed in border‐associated macrophages (BAM) [25] and together with Mrc1, Tgfbi, Ms4a7, and Lyve1, serves as a characteristic molecular marker of BAM [26]. PF4 expression has also been detected in microglia under both in vitro conditions and in models of neurodegeneration [27]. Interestingly, an increasing number of single‐cell transcriptomic studies have identified expanded populations of PF4^+^ macrophages in injured and diseased brain tissue [28]. Despite their increasing recognition, the functional significance of these PF4^+^ macrophages in CNS pathology remains poorly understood.

### 2.3 Function

PF4 is a secreted chemokine with diverse biological functions spanning coagulation, inflammation, angiogenesis, hematopoiesis, and fibrosis.

#### 2.3.1 Coagulation

PF4 was initially recognized for its role in coagulation and thrombosis, where it binds heparin‐like glycosaminoglycans, neutralizes their anticoagulant activity, and stabilizes thrombi [29]. PF4 is a key regulator in the pathogenesis of heparin‐induced thrombocytopenia (HIT) in which the PF4‐PF4 antibody‐heparin immune complexes activate platelets [29, 30]. More recently, PF4 has been shown to directly promote platelet activation and aggregation through the thrombopoietin receptor, cellular myeloproliferative leukemia protein (c‐Mpl) [31]. Collectively, these findings position PF4 as both a biomarker of platelet activation and a key effector of coagulation and thrombosis.

#### 2.3.2 Inflammation

PF4 exerts multifaceted immunomodulatory functions by regulating both innate and adaptive immune responses. It promotes the recruitment and activation of different immune cells, including monocytes, macrophages, neutrophils, and T cells. For example, PF4 forms heterodimers with CCL5 (RANTES), promoting monocyte adhesion to activated endothelium and enhancing vascular inflammation [14]. PF4 also stimulates differentiation of CD4^+^ T cells toward proinflammatory Th1 and Th17 phenotypes [32]. In addition, PF4^+^ macrophages have been implicated in immune remodeling in the tumor microenvironment by suppressing CD8^+^ T cell cytotoxicity and promoting T cell exhaustion [33]. Unlike classical Glu‐Leu‐Arg (ELR)^+^ CXC chemokines, PF4 lacks the ELR motif required for CXCR1/CXCR2‐mediated neutrophil chemotaxis and therefore does not directly recruit neutrophils. Instead, PF4 binds to the neutrophil surface through chondroitin sulfate, enhancing firm adhesion to activated endothelium. In the presence of co‐stimulatory cytokines such as TNF, PF4 promotes secondary granule release from neutrophils [34]. PF4 can also activate neutrophils through FcγRIIa signaling when complexed with anti‐PF4 antibodies, analogous to the mechanism underlying platelet activation in HIT [35]. PF4 binds to and compacts neutrophil extracellular trap (NETs), reducing their thrombogenic potential and associated endothelial injury [36]. A recent study suggests that PF4 can modulate the composition and molecular profiles of bone marrow cells, T cells, and natural killer cells within the aging immune system, thereby reducing age‐associated neuroinflammation [37]. Collectively, these findings suggest that PF4 functions as an important context‐dependent immunomodulatory chemokine that bridges innate and adaptive immunity across a range of pathological conditions. Notably, these immunoregulatory functions have been primarily demonstrated in vascular, thromboinflammatory, and systemic contexts. Whether PF4 exerts similar functions within the CNS remains largely unknown.

#### 2.3.3 Angiogenesis

PF4 is a potent inhibitor of angiogenesis. Its anti‐angiogenic effects are mediated primarily through CXCR3‐B, an alternatively spliced isoform of CXCR3 [38]. Through CXCR3‐B engagement, PF4 suppresses endothelial cell proliferation and migration, thereby counterbalancing pro‐angiogenic signals such as VEGF. Furthermore, PF4 forms heterocomplexes with IL8 and enhances its anti‐proliferative effects on endothelial cells [15].

#### 2.3.4 Neuroprotection

More recently, PF4 has emerged as a youth‐associated factor with neuroprotective properties. Systemic PF4 administration reduces age‐related hippocampal neuroinflammation and improves cognitive performance in aged mice [8]. Moreover, experimental work in CNS and retinal injury models shows that PF4 limits excitotoxic damage and secondary neuronal loss by dampening inflammasome activation and reshaping inflammatory signaling [39]. In addition, PF4 has also been identified as an exercise‐induced platelet‐derived exerkine that promotes neurogenesis and contributes to the cognitive benefits of physical exercise [40]. Consistent with these findings, lower circulating PF4 levels in patients with AD are associated with poorer cognitive performance, lower CSF Aβ42 levels, and higher CSF total tau concentrations [41], supporting a potential neuroprotective role for PF4 in aging and neurodegeneration.

#### 2.3.5 Other Functions

PF4 also helps maintain hematopoietic homeostasis by negatively regulating megakaryocyte and hematopoietic progenitor cell proliferation [42]. In addition, PF4 interacts with integrins (*αvβ3, αvβ5, α5β1*) to promote fibroblast differentiation and extracellular matrix deposition, implicating it in fibrosis and vascular remodeling [43].

## 3 Signaling Pathways Activated by PF4

### 3.1 PF4‐CXCR3 Signaling

PF4 regulates a wide range of cellular signaling pathways that underlie its diverse biological functions in different types of cells. Among its receptors, CXCR3 is best characterized and implicated in many of PF4's functions (Figure 2). As a G protein‐coupled receptor. CXCR3 activates Gi‐dependent signaling pathways, including MAPK and PI3K/AKT. In a murine model of ulcerative colitis, PF4/CXCR3 signaling enhanced intestinal inflammation through activation of MAPK and NF‐κB pathways in macrophages [44]. In monocytes and microglia, PF4 binding to CXCR3 suppresses Type I interferon responses and inhibits autophagy‐lysosomal degradation by activating mTOR, p38, and ULK1^Ser757^ while reducing LC3‐II expression, thereby facilitating viral replication during flavivirus infection [45]. PF4–CXCR3 signaling also contributes to tumor immune evasion, as β‐catenin‐activated hepatocellular carcinoma (HCC) cells secrete PF4 to recruit CXCR3^+^ myeloid‐derived suppressor cells (MDSCs), promoting an immunosuppressive microenvironment [46]. Interestingly, PF4‐CXCR3 signaling exhibits protective effects in neuronal cells. In this context, activation of AKT1/SLC7A11 and PI3K/AKT/Nrf2 pathways suppresses ferroptosis and oxidative stress following intracerebral hemorrhage, thereby reducing inflammation and promoting tissue repair [47, 48].

**FIGURE 2:**
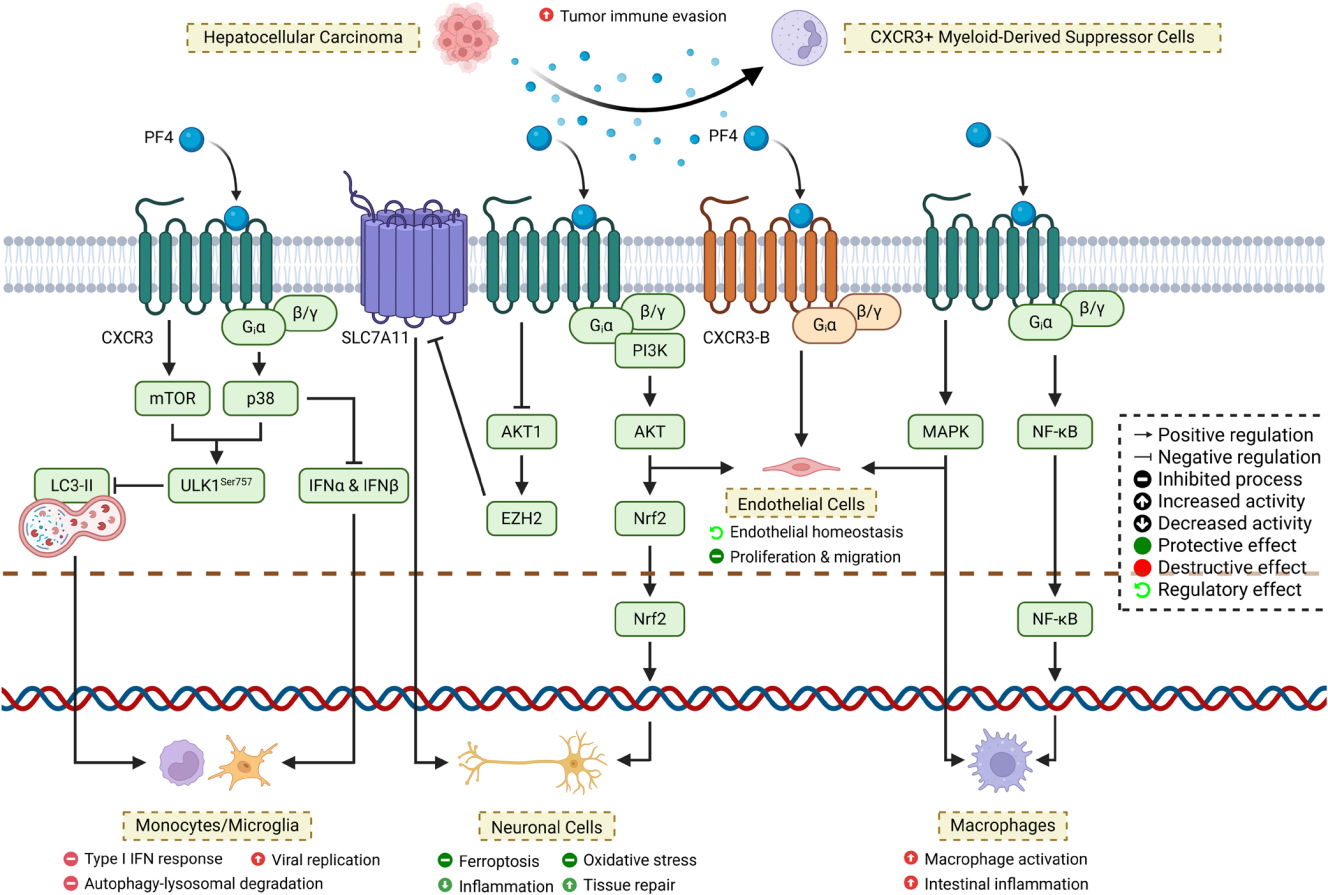
PF4/CXCR3 Interactions and downstream signaling pathways in different cell types. PF4 mainly binds to CXCR3 expressed on different cell types and modulates downstream signaling pathways. These signaling events lead to diverse cellular effects, including the regulation of ferroptosis, oxidative stress, inflammation, endothelial homeostasis, autophagy, fibrosis, viral replication, and tumor immune evasion. This figure was generated using BioRender.com.

### 3.2 Other Signaling

In platelets, PF4 binds to the thrombopoietin receptor c‐Mpl, leading to JAK2 activation and subsequent phosphorylation of STAT3 and STAT5, thereby promoting platelet aggregation [31]. In monocytes, PF4 rapidly induces reactive oxygen species production through activation of the p38 MAPK, Syk, and PI3K pathways, whereas its delayed effects on cell survival and cytokine production are mediated by ERK and JNK signaling [49] PF4 also activates NF‐kB signaling in inflammatory cells, contributing to the amplification of inflammatory responses [50]. In vascular endothelial cells, PF4 regulates multiple signaling pathways that coordinate endothelial activation, angiogenesis, and vascular remodeling. In human aortic endothelial cells, PF4 upregulates CD40 expression through SIRT1/NF‐κB/p65 pathway [51]. In human umbilical‐vein endothelial cells and human dermal microvascular endothelial cells, PF4 downregulates the expression of FLI1, a transcription factor essential for vascular development, maturation, and stability [52]. Furthermore, PF4 promotes endothelial‐to‐myofibroblast transition, leading to enhanced myofibroblast differentiation and extracellular matrix production, thereby contributing to fibrosis and vascular remodeling [43].

Collectively, these studies demonstrate that PF4 engages diverse receptor‐dependent signaling pathways to regulate hemostasis, inflammation, vascular homeostasis, tissue remodeling, and neuroprotection (Table 1). The downstream effects of PF4 are highly context dependent, varying according to receptor availability, target cell type, and disease state.

**TABLE 1:** Signaling pathways regulated by PF4.

Pathway	Species and cell type	Downstream mechanism of PF4 action	Biological effects	References
CXCR3/PI3K/AKT/Nrf2	Rat PC‐12 cells	↑ CXCR3 activation	↓ Neuronal ferroptosis & oxidative stress	[48]
		↑ PI3K & AKT phosphorylation		
		↑ Nrf2 nuclear translocation		
CXCR3/AKT1/SLC7A11	Rat PC‐12 cells	↑ CXCR3 activation	↑ Cell viability	[47]
		↓ AKT1 phosphorylation	↓ Ferroptosis & oxidative stress	
		↓ EZH2 activation	↓ Inflammatory response	
		↑ SLC7A11 expression		
NF‐κB signaling	Rat PC‐12 cells	↑ NF‐κB activation	↑ Inflammatory response	[50]
		↑ IL‐6, IL‐1β		
PI3K, Syk, p38 MAPK	Human monocytes	↑ PI3K, Syk, p38 MAPK activation	↑ ROS production	[49]
			↑ immediate innate immune responses	
Erk, JNK	Human monocytes	↑ Erk, JNK	↑ Protection against apoptosis	[49]
			↑ Pro‐inflammatory cytokine & chemokine	
c‐Mpl/JAK2/STAT3, STAT5	Human platelets	↑ c‐Mpl activation	↑ Platelet activation and aggregation	[31]
		↑ JAK2 activation		
		↑ STAT3 & STAT5 phosphorylation		
SIRT1/NF‐κB/p65	Human aortic endothelial cells	↓ SIRT1 expression	↑ Pro‐atherosclerotic endothelial inflammation	[51]
		↑ NF‐κB activation		
		↑ p65 acetylation & nuclear translocation		
		↑ CD40 expression		
SOCS3/STAT3	Human trophoblasts	↑ SOCS3 expression	↓ Trophoblast viability	[53]
		↓ JAK activation	↓ Proliferation, migration, invasion, EMT	
		↓ STAT3 phosphorylation	↓ Angiogenesis	
ITGA5‐FAK‐ERK	Human bone marrow mesenchymal stem cells	↓ ITGA5 activation	↓ Osteogenic differentiation	[54]
		↓ FAK activation		
		↓ ERK phosphorylation		
		↓ ALP, RUNX2, SP7		
		↓ SPARC		
KLF4 signaling	Human coronary artery smooth muscle cells	↑ KLF4 activation	↑ Cell proliferation	[55]
		↑ IL‐6, IL‐8		
LRP/NF‐κB/E‐selectin	Human umbilical vein endothelial cells	↑ LRP activation	↑ Endothelial activation	[56]
		↑ NF‐κB activation	↑ Leukocyte recruitment and adhesion	
		↑ E‐selectin expression and production	↑ Atherogenesis	

## 4 Effects of PF4 in Aging and NDs

PF4 has emerged as an active participant in the pathophysiology of NDs. Its effects appear to be highly context‐dependent, exerting both detrimental and protective actions depending on the disease stage and local microenvironment (Table 2).

**TABLE 2:** Clinical and experimental evidence for PF4 alterations and functions in aging and NDs.

Conditions	Clinical evidence	Experimental observations	References
Aging	Circulating PF4 levels decline gradually with age in humans and are associated with age‐related changes in platelet abundance and function	Recombinant PF4 administration improves cognitive performance in young and aged mice. PF4 mediates cognitive benefits associated with exercise and Klotho through regulating immune cell composition, reducing neuroinflammation, and enhancing hippocampal neurogenesis	[8, 57, 58, 59]
PND	Higher PF4 levels are associated with a reduced risk of PND in elderly patients after surgery	In aged mouse models of surgery‐induced cognitive impairment, reduced PF4 contributes to neuroinflammation. Recombinant PF4 treatment improves cognitive function	[60]
AD	Human studies report conflicting results. Some proteomic and bioinformatic analyses report increased serum PF4 in AD patients, whereas another cohort reports decreased serum PF4 levels, with lower PF4 associated with poorer cognition	Elevated cortical mRNA expression of PF4 is noticed in APP/PS1 transgenic mice	[41, 61, 62, 63]
PD	PF4 alterations are tissue‐dependent in human PD patients. Increased PF4 expression has been reported in peripheral blood mononuclear cells and serum exosomes, whereas reduced PF4 protein levels have been observed in the striatum	PF4 administration or brain‐specific PF4 overexpression improves motor function, reduces neuroinflammation, decreases α‐synuclein accumulation, and protects dopaminergic neurons in MPTP‐treated mice	[64, 65, 66, 67]
MS	Plasma PF4 levels are elevated in MS patients during remission and correlate positively with disease severity	Platelet‐derived PF4 accumulates around spinal cord neurons before disease onset in EAE mouse models. PF4 regulates CD4^+^ T cell differentiation in a concentration‐ and context‐dependent manner	[68, 69]
cSVD	PF4 is an independent predictor of radiological cSVD progression, particularly white matter lesion progression, in human patients	—	[70]

### 4.1 Age‐Related Cognitive Decline

Aging‐related cognitive deficits encompass a spectrum of diseases characterized by progressive memory loss, executive dysfunction, and other cognitive impairments. Aging is associated with cerebrovascular dysfunction, chronic low‐grade inflammation, mitochondrial impairment, and loss of proteostasis, all of which increase neuronal vulnerability [71]. Recent studies suggest that PF4 improves cognitive function by modulating the composition and molecular profiles of bone marrow cells, T cells, and natural killer cells within the aging immune system, thereby reducing age‐associated neuroinflammation [37]. Exercise‐induced platelet activation releases PF4, which has been implicated in promoting adult hippocampal neurogenesis and contributes to the pro‐neurogenic effects of exercise [57].

Klotho, a longevity‐associated factor, enhances cognitive function through the regulation of multiple signaling pathways [72, 73]. An untargeted proteomic analysis revealed that systemic Klotho administration activates platelets and increases the release of platelet‐derived proteins. Strikingly, pharmacological inhibition of platelet activation with oral aspirin and clopidogrel abolishes Klotho‐mediated cognitive benefits, demonstrating that these effects are platelet‐dependent. Subsequent work by Park et al. [58] identified PF4 as the most strongly upregulated factor following Klotho treatment and further confirmed that PF4 supplementation improved cognitive performance in both young and aged mice. Although PF4 is sufficient to reproduce several Klotho‐associated effects on synaptic plasticity and cognition, Klotho retains its cognitive‐enhancing effects in PF4‐deficient mice. These findings indicate that PF4 is not individually required for Klotho‐mediated cognitive enhancement and instead likely functions as one of several partially redundant platelet‐derived mediators that contribute to Klotho's effects. Consistent with these findings, circulating PF4 levels decline gradually with age in humans [59]. This decline is accompanied by age‐related alterations in platelet abundance and function. In humans, platelet counts remain relatively stable during middle age but tend to decrease in older adults, particularly at advanced ages. In contrast, platelet reactivity and agonist‐induced aggregation increase with age [74, 75]. Chronic low‐grade inflammation, increased reactive oxygen species production, and mitochondrial and metabolic remodeling may collectively contribute to the age‐associated hyperreactive and prothromboinflammatory platelet phenotype [75, 76]. PF4 might be a convergent mediator of the cognitive benefits associated with young platelet, exercise, and Klotho, establishing it as a youth‐associated neuroprotective signal in the aging brain.

### 4.2 Perioperative Neurocognitive Disorders (PND)

PND refers to a group of cognitive impairments that occur after surgery, predominantly in elderly patients. They are characterized by delayed neurocognitive recovery, typically occurring within 30 days postoperatively. Clinically, PNDs are characterized by impairments in memory, attention, executive function, and processing speed, which collectively impair daily functioning and quality of life [77]. Mechanistic studies have suggested that activation of the C3/C3aR signaling pathway and the consequent reduction in PF4 contribute to PND pathogenesis, whereas treatment with recombinant PF4 alleviates neuroinflammation and improves cognition in animal models [60].

### 4.3 Alzheimer's Disease

AD is a neurodegenerative disorder characterized by progressive memory impairment and cognitive decline, with β‐amyloid deposition and Tau pathology as its defining neuropathological hallmarks. Platelets are a major source of circulating Aβ1‐40 and can thereby exacerbate cerebral amyloid angiopathy, a key contributor to AD pathogenesis [78]. Beyond serving as a source of circulating Aβ, platelets from patients with AD exhibit enhanced basal activation, and experimental evidence supports a bidirectional interaction in which Aβ activates platelets, whereas activated platelets promote cerebrovascular Aβ aggregation, potentially establishing a feed‐forward loop between platelet dysfunction and amyloid pathology [79, 80]. The relationship between PF4 and AD is complex and potentially context‐dependent. Upon activation, platelets release chemokines including PF4 and CCL5, which promote leukocyte adhesion, inflammation, vascular injury, and cognitive deterioration, thereby accelerating AD progression [81]. Consistent with this concept, significantly elevated cortical mRNA levels of multiple chemokines, including PF4, were observed in APP/PS1 transgenic mice, a mouse model of AD [61]. Proteomic analysis shows increased serum PF4 levels in AD patients. A diagnostic model incorporating PF4, seven other proteins and demographic variables achieves high diagnostic performance [62]. Similarly, bioinformatic analysis of peripheral blood from AD patients identified PF4 as an upregulated hub gene associated with platelet activation, inflammatory signaling, and AD‐related pathways [63]. In contrast, a recent clinical study reported decreased serum PF4 levels in AD patients, with PF4 levels positively correlated with cognitive performance. In the same cohort, serum PF4 levels are positively correlated with CSF Aβ42 levels and negatively correlated with total tau levels in CSF. Moreover, combining PF4 with other CSF biomarkers improved diagnostic accuracy for AD [41]. These opposing reports of elevated versus reduced PF4 levels may reflect differences in disease stage, patient characteristics, biospecimen type, analytical platforms, or cohort composition. These discrepancies underscore the need for standardized and longitudinal studies to clarify the temporal dynamics of PF4 and to establish its utility as a reliable AD biomarker and as a therapeutic target.

### 4.4 Parkinson's Disease

PF4 alterations in PD appear to be tissue‐ and context‐dependent. Transcriptomic analysis of peripheral blood mononuclear cells identifies PF4 as an upregulated hub gene in PD [64], and PF4‐associated signals are similarly elevated in serum exosomes from PD patients [65]. In contrast, postmortem studies reported reduced PF4 protein levels in the striatum, with no significant change in the substantia nigra [66]. In addition, machine‐learning analysis of multiple whole‐blood transcriptomic datasets showed an overall downregulation of PF4V1/CXCL4L1, a PF4 homolog. It should be noted that, although PF4V1 is highly homologous to PF4, changes in PF4V1 expression cannot be directly interpreted as changes in PF4 itself [82]. Collectively, these findings suggest that PF4 alterations in PD may depend on tissue type, cellular origin, and analytical platform. It should be noted that these PF4 alterations occur in the context of broader platelet dysfunction in PD. Platelet hyperactivation and systemic hypercoagulability have been reported in PD [83], whereas studies examining platelet mitochondrial respiratory‐chain activity have yielded inconsistent findings, indicating a heterogeneous and context‐dependent metabolic state [84, 85, 86, 87].

Beyond these correlative findings, recent animal studies investigate the functional role of PF4 in PD. Intranasal administration of platelet‐derived extracellular vesicles enriched in PF4 improves motor function and preserves tyrosine hydroxylase expression in the substantia nigra of MPTP‐treated mice, a widely used experimental PD model. However, these vesicles also contained multiple neurotrophic, anti‐inflammatory, and antioxidant proteins. The observed benefits could not be specifically attributed to PF4 [88]. More direct evidence has recently shown that systemic PF4 administration or brain‐specific PF4 overexpression alleviated motor dysfunction, neuroinflammation, pathological α‐synuclein (α‐syn) accumulation, and dopaminergic neurodegeneration in MPTP‐treated mice [67]. This study supports a neuroprotective role for PF4 in experimental PD. Interestingly, one study shows that α‐syn, the key pathogenic protein in PD, is present in platelets and is associated with the membrane of secretory α‐granules [89]. Exogenous α‐syn dose‐dependently inhibits PF4 release from platelet α‐granules, an effect that is preserved even with PD‐associated α‐syn point mutations, suggesting a possible reciprocal interaction between platelet α‐syn and PF4 in PD pathogenesis [89].

### 4.5 Multiple Sclerosis (MS)

MS is an autoimmune demyelinating disease of the CNS characterized by multifocal neurological lesions, recurrent episodes of neurological dysfunction, and progressive disability. It is known that platelets participate in neuroinflammation during the initiation and progression of MS. At early stages of MS and experimental autoimmune encephalitis (EAE), a mouse model of MS, platelet degranulation releases 5‐hydroxytryptamine (5‐HT), PF4, and platelet‐activating factor (PAF), which promotes the differentiation of inflammatory T cells [32]. At later stages, platelets interacted with T cells via adhesion molecules to form platelet–T cell aggregates [32]. Clinical studies revealed that plasma PF4 levels were significantly elevated in MS patients during disease remission and positively correlated with disease severity. Notably, low‐dose aspirin significantly lowered plasma β‐thromboglobulin but did not reduce PF4 levels in these patients, suggesting that PF4 may derive not only from platelets but also from other sources [68]. Interestingly, platelet‐surface–bound PF4 fine‐tunes CD4^+^ T cell responses in a concentration, tissue context, and contact‐dependent manner. At low concentrations, PF4 enhances TGF‐β signaling to promote Treg differentiation and maintain Th17 homeostasis, whereas at high concentrations, PF4 blocks TGF‐β receptor signaling and suppresses immune regulation [90]. The contribution of platelet‐derived PF4 to neurodegeneration is further supported by findings from the EAE. Platelet‐derived PF4 and other proinflammatory platelet products accumulate around spinal cord neurons before the onset of clinical symptoms and extensive infiltration of T cells [69, 91]. Together, these findings offer new insights into the importance of PF4 in neurodegeneration after MS.

### 4.6 Cerebral Small‐Vessel Disease (cSVD)

cSVD is one of the most common vascular diseases of the brain [92]. A recent study in cSVD reports no significant differences in plasma PF4 levels. However, PF4 may be associated with cSVD pathogenesis when combined with neutrophil activation markers, supporting its potential as an auxiliary predictive indicator [93]. In a prospective study, PF4 levels are independently associated with radiological progression of cSVD. Subtype analysis further indicates that PF4 is particularly linked to the progression of white matter lesions [70]. These findings suggest that PF4 might be a marker of cSVD progression rather than a diagnostic biomarker.

Current evidence suggests that PF4 might be a multifaceted regulator of NDs, with effects that are highly dependent on disease context, stage, and microenvironment. In aging and PND, PF4 predominantly exhibits neuroprotective properties and is associated with improved cognitive function. In AD, PD, MS, and cSVD, however, PF4 appears to play more complex and sometimes opposing roles, reflecting differences in disease mechanisms and temporal dynamics. Inconsistent findings regarding PF4 in NDs also highlight the need for standardized longitudinal studies.

## 5 Targeting PF4 for ND Treatment

As current evidence suggests the importance of PF4 in NDs, therapeutic strategies targeting PF4 have been developed (Table 3).

**TABLE 3:** Therapeutic strategies targeting PF4 in neurodegenerative diseases.

Treatment strategy	Disease/model	Route	Dose	Timing	Mechanistic impact	Effect	References
Exogenous α‐synuclein	PD	Cell incubation	10 μM	5, 10, 30, 60 min before stimulation	Inhibiting ionomycin‐ & thrombin‐induced PF4 release from α‐granules in platelets	↓ PF4 secretion	[89]
	Human platelets						
Recombinant PF4	PND	i.v. Injection	500 ng	daily ×7 prior to surgery	↓ IL‐6 & TNF‐α, ↓ astrocyte & microglia activation	↓ Neuroinflammation	[60]
	Aged mice					↑ Cognitive function	
Recombinant PF4	Aging	i.v. Injection	500 ng	8 times over 24 days	Exerting pro‐youthful effects via CXCR3‐linked signaling	↓ Hippocampal inflammation	[8]
	Aged Mice					↑ Cognition	
Recombinant PF4	Aging	i.v. Injection	500 ng	Every third days over 24 days	Acting as an exerkine to mimic exercise‐induced benefits	↑ Hippocampal neurogenesis	[40]
	Aged Mice					↑ Cognitive function	

### 5.1 Suppress the Detrimental Effects of PF4

Targeting the deleterious inflammatory effect of PF4 may represent a therapeutic strategy to mitigate neuronal damage in NDs. As platelets are an important source of PF4, inhibiting platelet activation is expected to reduce PF4 release. Surprisingly, low‐dose aspirin treatment fails to lower PF4 levels in MS [68], suggesting that elevated PF4 may also originate from non‐platelet sources. Therefore, current approaches focus on broadly suppressing PF4 activity rather than targeting a specific cellular source.

RNA interference (RNAi)–based gene‐silencing approaches have shown promise in attenuating PF4‐mediated inflammation. In cellular models of intracerebral hemorrhage (ICH), shRNA‐mediated PF4 knockdown inhibited NF‐κB activation, decreased IL‐6 and IL‐1β production, and consequently alleviated inflammation‐induced cellular injury [50]. Disrupting PF4 interactions with other chemokines represents another potential anti‐inflammatory and neuroprotective strategy. In a mouse model of ischemic stroke, the synthetic cyclic peptide MKEY prevented the formation of PF4–CCL5 heteromers, reduced monocyte recruitment, decreased infarct size, and improved neurological outcomes [14]. Further studies are needed to evaluate the efficacy of PF4‐directed interventions in ND models.

### 5.2 Enhance the Protective Roles of PF4

Recent studies demonstrated that PF4 has rejuvenating effects in aging. Transfusion of young blood components or direct intravenous injection of recombinant PF4 into aged mice significantly reduces hippocampal inflammation and restores cognitive performance, mimicking the benefits of young plasma infusion [8]. Similarly, exercise‐induced platelet activation releases PF4, functioning as an “exerkine” to promote hippocampal neurogenesis and enhance memory. Intravenous PF4 administration alone is sufficient to recapitulate these neurogenic and cognitive benefits [40]. These findings highlight the therapeutic potential of PF4 for mitigating age‐related cognitive decline. However, whether these rejuvenating and neuroprotective effects can be extended to NDs remains unclear and warrants further investigation.

### 5.3 Clinical Trials Relevant to PF4 in Aging

Despite promising preclinical evidence supporting PF4‐mediated neuroprotection, clinical translation of PF4‐based therapies remains in its infancy. To date, no clinical trials have directly targeted PF4 for the treatment of NDs. However, several ongoing trials are investigating interventions that modulate biological processes closely related to PF4‐associated mechanisms in aging. A Phase 1 clinical trial (NCT07216781) is evaluating the safety of Klotho gene delivery and its potential effects on cognitive function in healthy adults. Circulating PF4 is included as a secondary outcome measure to assess platelet activation‐related changes and potential immune‐mediated coagulation effects following Klotho augmentation. In another Phase 1 study (NCT07285629) assessing combined Klotho and follistatin gene delivery in healthy old adults, circulating PF4 is measured alongside epigenetic aging biomarkers and multidomain cognitive outcomes to explore platelet‐related systemic responses and their potential associations with biological aging and cognitive function. A separate ongoing clinical study (NCT07731451) is investigating the metabolic mechanisms that may support brain resilience during aging, including their potential neurotrophic and cognitive benefits. In this study, PF4 concentrations are measured as a brain‐specific biomarker to characterize lactate‐associated changes in brain function. Results from all three trials have not yet been reported.

## 6 Challenges in Research on PF4 in NDs

The major challenge in defining the role of PF4 in NDs lies in its context‐dependent activities. In pathological settings, PF4 promotes monocyte/macrophage and neutrophil activation [18, 35], amplifying neuroinflammation and neuronal injury [14, 50]. Notably, much of the evidence supporting these detrimental effects of PF4 comes from acute brain injury models, including ICH and ischemic stroke [14, 47, 48, 50]. Whether these findings can be extrapolated to chronic NDs remains unclear. In contrast, under physiological or aging‐related conditions, PF4 attenuates hippocampal neuroinflammation, enhances adult neurogenesis, and improves cognitive function. So far, evidence linking PF4 to NDs remains largely correlative, with limited functional validation.

Another unresolved question is the source and route of PF4 action within the CNS. Although platelet α‐granules constitute the major circulating reservoir of PF4, other cell types, including monocytes, macrophages, activated T cells, and possibly CNS‐resident cells, can also express or release PF4 under pathological conditions. The relative contributions of peripheral versus CNS‐derived PF4 remain unclear. It is uncertain whether circulating PF4 directly enters the CNS or instead exerts its effects indirectly through neurovascular interaction or modulating peripheral immune signaling.

Several challenges remain in translating PF4 research into clinical applications despite promising preclinical findings. Conventional antiplatelet agents fail to significantly reduce PF4 levels [68, 94], and more selective interventions, including RNA interference, blocking antibodies, and small‐molecule inhibitors—remain in the preclinical stage [43, 45, 58, 95, 96]. To date, no clinical interventional trials have specifically targeted PF4 in NDs. Given PF4's central role in coagulation, systemic manipulation of PF4 may increase the likelihood of bleeding or thrombosis. In addition, PF4 can bind to multiple chemokine receptors, including CXCR3 [44], CCR1 [18], and CXCR2 [15]. Conversely, CXCR3 can be activated by various chemokines, including PF4, CXCL9 [97], CXCL10 [98], and CXCL11 [99]. Therefore, the regulation on PF4 activity may have impacts on multiple chemokine‐receptor systems, resulting in unexpected effects on immune responses. These factors must be carefully considered when designing PF4‐targeted pharmacological interventions.

## 7 Conclusion

PF4 is increasingly recognized as a regulatory molecule in NDs. Current evidence demonstrates that PF4 exerts multifaceted effects under different disease conditions. Future research should focus on elucidating the sources, spatiotemporal dynamics, and functions of PF4 in different NDs. Therapeutically, precision modulation is required to suppress PF4's pathological effects and enhance its beneficial roles. PF4 might become a biomarker and a potential therapeutic target to diagnose and treat NDs.

## Funding

This work was supported by National Institutes of Health, NS131169, NS124673 U.S. Department of Veterans Affairs, BX006534, RD003028.

## Conflicts of Interest

Xiaoming Hu is the Associate Editor of CNS Neuroscience and Therapeutics and a co‐author of this article. They were excluded from editorial decision‐making related to the acceptance and publication of this article. Editorial decision‐making was handled independently by the other editor to minimize bias.

## Data Availability

Data sharing not applicable to this article as no datasets were generated or analysed during the current study.
